# The soluble ST2 levels in patients with depression and comorbid heart failure

**DOI:** 10.1192/j.eurpsy.2022.314

**Published:** 2022-09-01

**Authors:** A. Sikora, S. Fedorov, M. Vynnyk, M. Pustovoyt, O. Pityk, J. Bezuk

**Affiliations:** 1 Ivano-Frankivsk National Medical University, Psychiatry, Narcology And Medical Psychology, Ivano-Frankivsk, Ukraine; 2 Ivano-Frankivsk National Medical University, Postgraduate Internal Medicine, Ivano-Frankivsk, Ukraine

**Keywords:** Heart Failure, Nurobiological Factors, Depression, comorbidity

## Abstract

**Introduction:**

Depression in HF has become a major issue as the burden of HF has continued to increase, and many studies have suggested poorer outcomes in HF patients reporting depression. The prevalence of major depression in HF is about 20–40 %, which is 4–5 % higher than in the normal population. Soluble ST2 is involved in multiple pathogenetic pathways including cardiac strain, inflammation, and myocardial necrosis with remodeling.

**Objectives:**

The purpose of study was to assess the predictive effect of soluble ST2 (sST2) and depressive symptoms in patients with ischemic HF

**Methods:**

It this observational cross-sectional trial 129 patients with ischemic HF FC II-IV by New York Heart Association and depression were investigated. The diagnosis was verified by laboratory and instrumental methods according to European Society of Cardiology recommendations (2016). Depressive symptoms were evaluated by the Hospital Anxiety and Depression Scale. The ST2 level in blood serum was detected by ELISA method. Statistical analyses were performed using the Statistica 12 (StatSoft, Tulsa, OK, USA).

**Results:**

The prevalence of depression increases with NYHA functional class. With decreasing ejection fraction of left ventricle, levels of sST2 were gradually increased (P for trend < 0.001), as well as the prevalence of depressive symptoms (P for trend < 0.01). Multivariate Cox regression analysis demonstrated that depressive symptoms and elevation of sST2 were both independent predictors of all-cause mortality and HF-related hospitalization.

**Conclusions:**

The serum levels of sST2 and depressive symptoms were independent and additive predictors of all-cause mortality and heart failure-related hospitalization in patients with ischemic HF.

**Disclosure:**

No significant relationships.

